# Is the Achievement Motive Gender-Biased? The Validity of TAT/PSE in Women and Men

**DOI:** 10.3389/fpsyg.2017.00181

**Published:** 2017-02-15

**Authors:** Nicole Gruber

**Affiliations:** Department of Psychology, Universität RegensburgRegensburg, Germany

**Keywords:** implicit motive, achievement, Thematic Apperception Test, gender, sex, validity

## Abstract

In picture story exercises like the Thematic Apperception Test (TAT; Heckhausen, 1963), different pictures are presented to a person with the instruction to create a story using the scenes portrayed in the image. It is assumed, that people identify themselves with the people in the images and project their unconscious motives (e.g., achievement motive) onto them. As the TAT shows only men in the pictures, critics claimed the test is gender-biased; assuming women cannot identify with men in pictures. However, it was not assessed, whether female protagonists of the picture really trigger the same achievement motive as men. Therefore, two studies were conducted to address the gender difference and validity of the TAT using a version with only men in the pictures (study 1) or only women in the pictures (study 2). The results shows that the original TAT of Heckhausen is a valid instrument for women and men, but the modified version with only women in the pictures cannot validly measure the achievement motive in the male sample.

## Introduction

### Motivation and its measurement

The achievement motive is a relevant factor for personal selection and educational psychology. Therefore, it is important to ensure that it is measured validly and independently of participant's gender. McClelland (1953) defined the achievement motive as “competing with a standard of excellence” (p. 110). Later this standard of excellence was interpreted as to do something as good as possible to get success (= hope of success; HS) or to avoid mistakes (= fear of failure; FF). Atkinson (1966) used this differentiation in his expectancy-value-theory and expected that people with high HS-scores prefer moderate tasks and those with high FF-scores choose easy or difficult tasks. The independence of these two motivational components HS and FF is important in new motivational models (e.g., the quadripolar model of Covington and Roberts, 1994). Furthermore, implicit (unconscious) and explicit (conscious) motives can be distinguished. Implicit motives develop in early childhood, remain mostly stable into old age and are related to long-term and spontaneous behavior. On the other hand, explicit motives are a little more modifiable and lead to planned and short-term behavior (McClelland et al., 1989). Spangler (1992) reported in a meta-analytical review that only high implicit (not explicit) achievement motive is related with long-term success and productivity. Therefore, implicit motives are very important. After Heckhausen (1963) analyzed HS and FF, he found that especially high implicit HS leads to better grades. For implicit and explicit motives are different, both are assessed using different measurements. For measuring the explicit motives questionnaires are used, implicit motives are assessed with the Thematic Apperception Test (TAT; Morgan and Murray, 1935). Here, people get presented several pictures of people in distinctive situations. The rationale behind this procedure is that persons identify themselves with the persons in the pictures and project their own unconscious motives onto them. Morgan and Murray (1935) noticed a substantial problem associated with this concept: there are specific themes where women can only identify themselves with female protagonists and men only with male protagonists (e.g., the relationship between father and son or mother and daughter). Although there is no evidence that achievement themes are also gender specific, McClelland et al. (1989) took the factor “gender” into account in the development of his achievement TAT/PSE. He created pictures with same numbers of women and men, for example the picture “architect at the desk” for men, and the picture “two women in lab coats in laboratory” for women (Smith, 1992). Heckhausen (1963) revised the PSE of McClelland et al. (1958) and translated it into German. He adopted from McClelland et al. (1958, 1989) that the people have 5 min to tell a story for each of the five pictures based on the questions, who the people on the picture are, what they are thinking and feeling, what happened before and how it will go out. He also used similar pictures for stimulating hope of success (HS) and developed new pictures for fear of failure (FF) inspired by the idea that FF can be excited by an authority person (like the director or a teacher). Afterwards these protocols are analyzed by his coding-system. The unique component of his measurement is that it assesses HS and FF independently (see Covington and Roberts, 1994). So the assumptions of the expectancy value theory could validly be assessed with this implicit measure (Atkinson, 1966). Therefore, it was translated into English and widespread by Schultheiss (2001), provide and offered an important basis for further content-coding systems (Pang, 2010; Ramsay, 2014). Compared with another coding-system for the achievement motive, the coding system of Winter it is more specific and captures the achievement motive more detailed (Strasser, 2010).

### Reasons for gender differences

But Heckhausen neglected the factor “gender” for his TAT/PSE and created six pictures representing only men. Critics claimed the test could not measure the motives of women validly, for women are not able to identify themselves with men (Heckhausen and Heckhausen, 2010; Pang, 2010). Indeed, there is some evidence that people normally identify themselves with persons of same gender in the pictures, so the TAT is also used in gender and sexuality research (Silverstein, 2016). The general reason therefore is that the protocols represent the inner world of a person and the specific stimulus (the card) evokes the motive according to peoples' personal experiences (Mischel and Shoda, 1995). So if a woman sees women in the picture doing work, that she also often does it probably would more stimulate the achievement motive than watching men working something, because the first situation is more similar to own experiences. So some researcher recommend to portray both genders in the pictures that both men and women have the same possibility to project their unconscious wishes onto the persons in the pictures (resp. Fodor and Carver, 2000; Trash and Elliot, 2002; Duncan and Peterson, 2010). But a next problem occurs that people are more often seen as couple than as colleagues. Another strategy in handling the gender problem is to present the participant pictures with neither women nor men on it. In the OMT (Kuhl and Scheffer, 1999), a variation of the TAT/PSE, instead of detailed photography's only schematic sketches were used without any sexual signs like a beard. However, no one ever verified that the gender of the persons in the pictures really influences the achievement motive of participants (Teglasi, 2010; Silverstein, 2016). Indeed, some studies indicate that there is no gender-effect for the TAT/PSE (Chusmir, 1985; Katz et al., 1993). Aronow et al. (2013) pointed out the interpretation of a woman's TAT/PSE story with a male protagonist may be confounded by gender-specific attitudes. Talbot (2000, cited in Eisenchlas, 2013) claimed women to be more associated with affiliation and sociality than with achievement and control. Natinsky (2004) assumed, citing a study of Worchel et al. (1990), pictures of women to trigger more fear-related content, because women are more fear motivated. Stewart and Chester (1982) stated the achievement motive to be “consonant only with male sex roles” (p. 181) and therefore analyzed 14 studies but did not find a gender related effect. Also other researchers reported no influence of gender on the overall achievement motivation score or even on the same pictures (e.g., Schultheiss and Brunstein, 2001; Tuerlinckx et al., 2002; Pang and Schultheiss, 2005; Langan-Fox and Grant, 2006).

However all studies focus on descriptive differences, they cannot answer the question, if Heckhausens TAT/PSE is valid for women and men and how the gender of the protagonist in the pictures influences the validity of the TAT/PSE.

As the test is widespread and used over decades of years, the main question of the paper is, whether there really is a gender-effect for the TAT/PSE. Beyond this it is interesting to assess if the stimulation of a motive still works when the person is solely confronted with pictures of opposite gender. But the assumption of projection is, that even if the scores of women and men do not differ in measurements, the validity for women could be lower as they cannot identify with men on pictures. So if gender-specific attitudes are important the TAT/PSE of Heckhausen (1963) could be valid also for women as men are more associated with achievement.

### Assumptions

The initial assumption is that the TAT/PSE has no gender-related influence, the scores of HS and FF should not differ in both genders even on the same pictures. If there is a gender-influence, the motives of participants with the same gender, as represented in the pictures, should be higher than those with opposite gender. There should also be no differences in the validity-criteria between women and men, regardless if women or men are represented in the pictures.

Here, are some assumptions how a valid TAT/PSE should measure:

1. First the criterion of discriminant validity states that measurements of implicit and explicit motives (e.g., questionnaires) are independent. This is reported in many studies and meta-analysis (McClelland et al., 1989; Spangler, 1992; Brunstein and Hoyer, 2002; Schultheiss et al., 2009; Köllner and Schultheiss, 2014).
2. Construct validity assumes that HS and FF are two independent factors of achievement motive and their inter-correlation should be low (Heckhausen, 1963; Covington and Roberts, 1994).
3. Another validity method is criterion validity. Therefore, the expectancy-value theory of Atkinson (1966) was used: It is assumed that people with high HS prefer moderate tasks and those with high FF-scores choose easy or difficult tasks (Heckhausen, 1963).
4. Furthermore, implicit achievement motives should be related to study grades (Heckhausen, 1963; Spangler, 1992) and study effort (e.g., learning time) as it energizes the achievement behavior (McClelland et al., 1989).
5. Next it should correlate with learning behavior in the way that HS leads to positive efficient learning and FF to negative learning behavior like for example self-handicapping (Chen et al., 2009; Gruber, 2014; Haghbin et al., 2012; De Castella et al., 2013).

## Study 1

In the first study it is researched whether the original TAT/PSE of Heckhausen (1963) consisting of male pictures is a valid measure for both men and women. There should be similar low correlations between the motive-scores of self-reported questionnaires and the TAT/PSE (McClelland et al., 1989) in both genders. The correlations of implicit motive-scores with outcome variables like learning time and learning behavior, study grade and task-choice performance should also not differ for women and men (Atkinson, 1966; Spangler, 1992; De Castella et al., 2013).

### Materials and methods

#### Design and participants

Before the testing, required sample size was calculated for women and men using g-power. It should be at least 100 so that the power of pearson-correlation as well as *t*-test is high enough (effect size = 0.40, α-level = 0.05, power = 0.95).

One Hundred and fifty people (75 women and 75 men) took the test. The age of women was from 18 to 32 (*M* = 21.81, *SD* = 2.42), the age of men from 18 to 38 (*M* = 22.81, *SD* = 3.47). All were enrolled in different subjects at the University of Regensburg in different semesters (*M*_women_ = 3.78, *SD*_women_ = 2.25; *M*_men_ = 4.00; *SD*_men_ = 2.07). All procedures performed in the two studies involving human participants were in accordance with the ethical standards of the institutional and/or national research committee and with the 1964 Helsinki declaration and its later amendments or comparable ethical standards.

#### Materials

Materials were a questionnaire of demographic data (code, age, sex, semester, subject, graduation, overall grades, time of learning during semester, while holidays and before examinations, experience of typing, and handwriting). As measurements of the explicit system the SELLMO (Spinath et al., 2012) and the TAT-Q (Gruber, 2014) were used. The scales of learning and achievement motivation (SELLMO; Spinath et al., 2012) is a self-attributed questionnaire that assesses the four goals “achievement goals,” “avoidance goals,” “learning goals,” and “effort-avoidance goals” with 31 items on a 0–5 Likert-scale, the goals were used in this study. The TAT-Q is a questionnaire for hope of success and fear of failure based on the 11 content-coding categories of Heckhausen (1963). It was composed on the idea of Schultheiss et al. (2009) who claimed, that even measuring the same categories questionnaire and implicit measures will show different results. Therefore, Schultheiss et al. (2009) developed based on the coding-categories of Winter (1993 cited in Schultheiss et al., 2009) the PSE-Q. Next there was used a task-choice performance test (TCPT; Gruber, 2014) including nine drawing tasks of ascending difficulty (three easy, three medium, three hard). This test was made for checking the task-choice-performance as claimed by Atkinson (1966) and works as following: First people have to rate their ability in drawing tasks like “The house of St. Nicholas” from low (0), to medium (1), and high (2). Thereafter they must open the test-book and have to choose three out of the nine possible tasks, which they would do. Based on this choice the two scores were assessed: correct choices and distance. Correct choice is, when someone exactly chooses tasks fitting his/her ability e.g., if someone with medium ability chooses the medium-difficult tasks 4, 5, and 6 three correct choices were counted. Distance means the difference of difficulty of the tasks e.g., in the upper example the distance is 2 (1 + 1), because 6 − 5 = 1 + 5 − 4 = 1.

In a last part there was the questionnaire for learning behavior (QLB; Gruber, 2014) that measured positive learning behavior like “before a test I repeat carefully the things, that could occur in the test” and negative self-handicapping behavior like “before the test I go out to party and drink.” The main material were the six pictures of Heckhausen (1963), a clock and sheets of paper.

#### Procedure

The participants were recruited with fliers, posters, emails, and within courses. Each session was held in a university room, the test lasted about 1 h. People first participated on the TAT/PSE. The six pictures of Heckhausen (1963) were presented for 20 s to the people, than they got 5 min time to write who they think the persons in the pictures are, what they are thinking and feeling, what they are doing before and how it comes out. After the TAT, they completed the demographic data, the TCPT, then the TAT-Q, the QLB, and the SELLMO. If participants studied psychology they got credits for taking the test, otherwise they get only candy as thank you.

#### Analysis

First the stories were scored for the implicit need for achievement by two trained coders using the Heckhausen scoring system (1963; with an additional category proposed by Breidebach, 2012), which allows separated coding of hope of success (HS) and fear of failure (FF). Heckhausen (1963; English language translation by Schultheiss, 2001) assessed these two motivational components by six categories: the instrumental activities to achieve success resp. to avoid failure (IS/IF), the affective state (A+/A−), the expected goal state (ES/EF), the need for achievement (NS/NF), failure (F), and the achievement theme (ST/F). Breidebach (2012) suggests a new category he called “sureness of success” (*E*_*SG*_), which should be the pedant of failure in the FF category, so this aspect was also coded. The inter-rater-agreement of all studies assessed with the *a*_*d*_-coefficient by Kreuzpointner et al. (2010) and Pearson correlations (given in brackets) was *a*_*d*_ = 0.996 for HS (*r* = 0.93) and *a*_*d*_ = 0.998 for FF (*r* = 0.89), which is in both cases above the 95% level. Also the intra-rater-agreement in a delay of 4 weeks was measured: for HS between *a*_*d*_ = 0.961 (*r* = 0.89) and *a*_*d*_ = 0.997 (*r* = 0.92) and for FF between *a*_*d*_ = 0.941 (*r* = 0.87) and *a*_*d*_ = 0.999 (*r* = 0.95). Being a very strict measure, the high *a*_*d*_-coefficients indicate high objectivity.

For criterion and construct validity two-tailed pearson correlations between the HS- and FF-scores and the different tests were calculated. To find out whether these correlations differ in men and women a two tailed z-test for independent pearson correlation was used. The correlations were transformed according to Fisher (1915) and assessed regarding their difference. To test the general difference for motives scores for each picture a *t*-test was calculated, using Cohens d as effect size.

### Results

**Figure 1:**
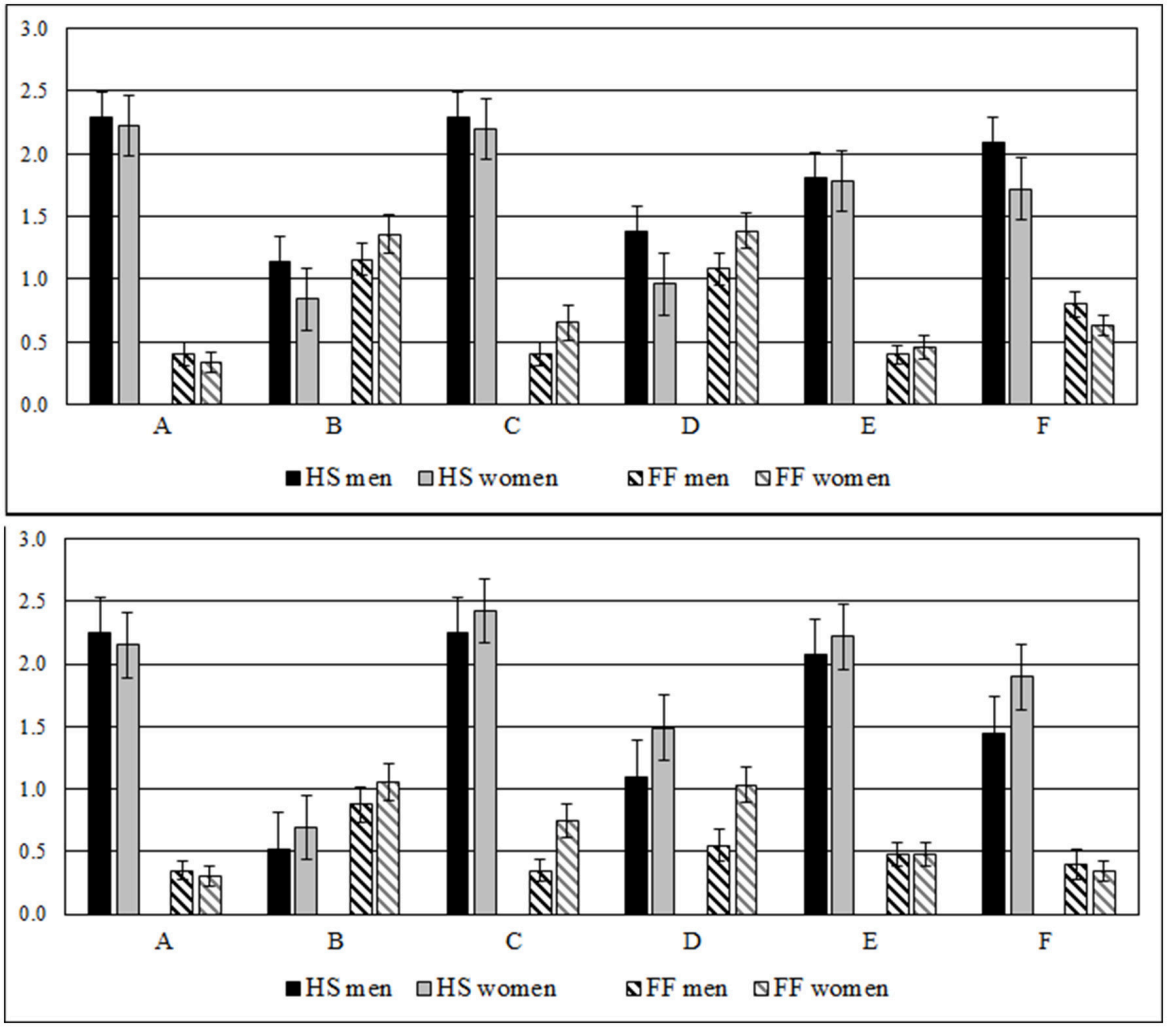
**(Upper)** Difference of men (*N* = 75) and women (*N* = 75) for the score of hope of success (HS) and fear of failure (FF) per picture (A to F) using the original pictures of Heckhausen (1963). **(Below)** Difference of men (*N* = 40) and women (*N* = 60) for HS and FF using the modified female pictures of study 2.

As obvious in Figure 1 both genders show similar scores for hope of success- and fear of failure-scores. So the HS scores for both genders of picture A [*M*_men_ = 2.29, *SD*_men_ = 1.27, *M*_women_ = 2.23, *SD*_women_ = 1.29, *t*_(148)_ = 0.32, *p* = 0.75, *d* = 0.32], picture B [*M*_men_ = 1.15, *SD*_men_ = 1.00, *M*_women_ = 0.84, *SD*_women_ = 1.10, *t*_(148)_ = 0.08, *p* = 0.67, *d* = 0.01], picture C [*M*_men_ = 2.29, *SD*_men_ = 1.40, *M*_women_ = 2.20, *SD*_women_ = 1.28, *t*_(148)_ = 0.43, *p* = 0.67, *d* = 0.07], and picture E [*M*_men_ = 1.81, *SD*_men_ = 1.41, *M*_women_ = 1.79, *SD*_women_ = 1.34, *t*_(148)_ = 0.12, *p* = 0.09, *d* = 0.02] were the same. Only for picture D [*M*_men_ = 1.39, *SD*_men_ = 1.37, *M*_women_ = 0.96, *SD*_women_ = 1.12, *t*_(148)_ = 2.08, *p* = 0.04, *d* = 0.34] and picture F [*M*_men_ = 2.09, *SD*_men_ = 1.21, *M*_women_ = 1.72, *SD*_women_ = 1.06, *t*_(148)_ = 2.00, *p* = 0.05, *d* = 0.33] men show higher HS scores than women.

The FF-scores generally did not differ at all for women and men, not for picture A [*M*_men_ = 0.40, *SD*_men_ = 0.79, *M*_women_ = 0.33, *SD*_women_ = 0.62, *t*_(148)_ = 0.58, *p* = 0.56, *d* = 0.10], picture B [*M*_men_ = 1.16, *SD*_men_ = 0.13, *M*_women_ = 1.36, *SD*_women_ = 1.23, *t*_(148)_ = −1.06, *p* = 0.29, *d* = 0.17], picture C [*M*_men_ = 0.04, *SD*_men_ = 0.82, *M*_women_ = 0.65, *SD*_women_ = 0.86, *t*_(148)_ = −1.84, *p* = 0.07, *d* = 0.30], picture D [*M*_men_ = 1.08, *SD*_men_ = 1.15, *M*_women_ = 1.40, *SD*_women_ = 1.24, *t*_(148)_ = −1.57, *p* = 0.12, *d* = −0.26], picture E [*M*_men_ = 0.40, *SD*_men_ = 0.66, *M*_women_ = 0.45, *SD*_women_ = 0.81, *t*_(148)_ = −0.44, *p* = 0.66, *d* = −0.07], and picture F [*M*_men_ = 0.80, *SD*_men_ = 0.87, *M*_women_ = 0.63, *SD*_women_ = 0.93, *t*_(148)_ = 1.18, *p* = 0.24, *d* = 0.19].

#### Construct validity

The first question refers to the relationship between hope of success (HS) and fear of failure (FF). The correlations between the two factors are with −0.20 (*p* < 0.01) in the male sample and −0.30 (*p* < 0.01) in the female sample not significantly different. (*Z* = 1.02, *p* > 0.05).

As obvious in Table 1 the correlations between the implicit motives and the motive scales of SELLMO and TAT-Q are similar low for men (*r* = −0.10–0.12) and women (*r* = 0.15–0.19). The only difference was, that women with high implicit HS show less avoidance of effort (*r* = −0.26, *p* < 0.05) and low self-reported HS (*r* = −0.28, *p* < 0.05). The last correlation differed between both genders (*Z* = 2.33, *p* < 0.05): In the male sample the self-reported HS and the implicit HS are almost independent (*r* = 0.10, *p* > 0.05).

**Table 1:** **Discriminant validity of TAT/PSE with male pictures for men (*N* = 75) and women (*N* = 75)**.

	**Hope of success**				**Fear of failure**			
	**Men**	**Women**	***Z***	***p***	**Men**	**Women**	***Z***	***p***
SELLMO_LG	0.13	0.03	0.48	0.63	0.12	−0.02	0.67	0.50
SELLMO_AV	0.03	−0.13	0.76	0.44	0.06	0.05	0.05	0.96
SELLMO_VG	0.05	−0.13	0.86	0.38	0.10	−0.02	0.57	0.56
SELLMO_AG	−0.09	−0.26^*^	0.83	0.41	0.03	0.15	−0.57	0.57
TAT-Q_HS	0.10	−0.28^*^	1.84	0.07	−0.10	0.19	−1.39	0.17
TAT-Q_FF	−0.01	−0.15	0.67	0.50	0.02	0.13	−0.52	0.60

SELLMO, Scales of learning and achievement motivation; LG, learning goals; AG, approach goals; VG, avoidance goals; AV, effort-avoidance goals; HS, hope of success; FF, fear of failure.

* *p < 0.05*.

#### Criterion validity

In a further step, the relationship between TAT-scores and outside criteria was researched. The results are provided in Table 2. First, it was looked how the implicit motives can correlate with task-choice-performance, so that people with high implicit HS choose medium difficult (correct) task and people with high implicit FF should choose very difficult or easy but not medium difficult tasks. The numbers of correct choices was negatively related to FF in the male (*r* = −0.26, *p* < 0.05), and female sample (*r* = −0.34, *p* < 0.05, *Z* = 1.86, *p* > 0.05). The distance between choices was independent from HS or FF.

**Table 2:** **Criterion validity of TAT/PSE with male pictures for men (*N* = 75) and women (*N* = 75)**.

	**Hope of success**				**Fear of failure**			
	**Men**	**Women**	***Z***	***p***	**Men**	**Women**	***Z***	***p***
TCPT_CC	0.04	0.13	−0.43	0.67	−0.26^*^	−0.34^**^	0.42	0.67
TCPT_DC	−0.21	−0.20	−0.05	0.96	0.01	−0.03	0.19	0.85
LT general	−0.23^*^	0.04	1.3	0.19	0.04	0.03	0.05	0.96
LT before tests	0.23^*^	0.13	0.49	0.62	−0.06	−0.05	−0.05	0.96
LT during holidays	−0.24^*^	0.19	−2.07	0.04	0.19	−0.08	1.29	0.20
Overall grade	−0.36^**^	−0.29^*^	−0.37	0.71	−0.29^*^	−0.14	−0.75	0.45
QLB_positiv	0.19	−0.05	1.15	0.25	−0.02	0.10	−0.57	0.57
QLB_negativ	−0.19	−0.20	0.05	0.96	0.24^*^	−0.01	−1.11	0.27

TCPT, Task-choice-performance-test; CC, correct choices; DC, distance of choices; LT, learning time; QLB, questionnaire of learning behavior.

* p < 0.05,

** *p < 0.01*.

Regarding learning times it is shown that general learning time (*r* = −0.23, *p* < 0.05) and learning time before examinations (*r* = 0.23, *p* < 0.05) were only related to the implicit HS in the male sample but not in the female sample. Women with high HS prefer a little more to learn during the holidays (*r* = 0.19, *p* > 0.05) than men do (*r* = −0.24, *p* < 0.01, *Z* = 2.62, *p* < 0.05).

For both genders HS correlates with better grades in the male (*r* = −0.36, *p* < 0.05) and female sample (*r* = −0.29, *p* < 0.05, *Z* = 0.47, *p* > 0.05). The same can be reported for FF it also correlates more strongly with grade in the male sample (*r* = −0.29, *p* < 0.05) than in the female sample (*r* = −0.14, *p* < 0.05; *Z* = 0.55, *p* > 0.05). Regarding positive and negative learning behavior it is shown that positive learning behavior correlates with both HS and FF in the female (*r* = −0.05–0.10) in the male sample very low (*r* = 0.02–0.19). Especially men with high FF show high negative learning behavior like going out before an examination (*r* = 0.24, *p* < 0.05) compared to women (*r* = −0.01, *p* > 0.05).

#### Brief discussion

First it can be reported, that on four out of six pictures the motive-scores women and men on the pictures does not differ much. This result is similar to others (see Schultheiss and Brunstein, 2001; Tuerlinckx et al., 2002; Pang and Schultheiss, 2005; Langan-Fox and Grant, 2006). It is only shown that men show higher HS scores in pictures that were made to measure FF (picture D and F). This could be a hint that for men the male authority in the pictures is not as threatening as for women. The validity of the measurement is the same: The independence between implicit and explicit motives is attested for both genders, although the implicit HS of women correlates more with the explicit HS than in the male sample. HS and FF could not be declared as independent factors in both samples. But the relationship of specific task-choice performance and the correlations between the motive-scores and grades could be verified in both samples. The correlations of motive-scores and external learning criteria (learning behavior and learning time) are more given in the male sample than in the female sample. To look whether gender really plays no role for the measurement of achievement motive, in the next study a TAT/PSE with only female protagonists in the pictures was presented to both genders.

## Study 2

The second study examined the validity of a TAT/PSE with only female protagonists in the pictures, representing the same situations as in study 1. With this design it is easier to assess the gender-effect of the TAT than for example having a couple in the picture. The assumptions were the same as in study 1, so that the TAT/PSE-scores should be similar and not correlate with self-reported questionnaires (McClelland et al., 1989) for women and men. The correlations between the TAT/PSE-score and the outcome variables learning time, learning behavior, grade and task-choice performance should be similar to those in study 1 (Atkinson, 1966; Spangler, 1992; De Castella et al., 2013).

### Materials and methods

#### Design and participants

Hundred people (60 women and 40 men) took the test. The age of men range from 19 to 30 years (*M* = 22.12, *SD* = 1.76), that of women from 19 to 26 years (*M* = 21.15, *SD* = 2.35). All were enrolled in different subjects at the University of Regensburg in different semesters (*M*_men_ = 4.45; *SD*_men_ = 1.56; *M*_women_ = 3.50, *SD*_women_ = 1.46).

#### Materials

The material was the same as in study 1 only the pictures of the TAT/PSE of Heckhausen (1963) were modified. So photographs were collected for women representing the same situations as the men in the original pictures. Basic characteristics like age, facial expression, period of the 60th was also the same. Altogether the pictures were: a woman on the desktop, a woman before the directors' room, a woman in front of a female teacher. The pictures “two men on a workbench” and “in front of the instructor” were replaced by “two women in the kitchen” and “two women in the laboratory” (Smith, 1992).

#### Procedure

The procedure was the same as in study 1. All participants first took the TAT with respect to the standardized instruction of Heckhausen (1963). Then they got the same questionnaires as in study 1: SELLMO (Spinath et al., 2012), TAT-Q, questionnaire of learning behavior and the task choice performance test (Gruber, 2014).

#### Analysis

The stories were scored for the implicit achievement motive as in study 1 all scores were satisfying (see study 1). Similarly to study 1, the criterion and construct validity was assessed with pearson correlations between the HS- and FF-scores and their correlation with several tests. To find out if these correlations differ between men and women, a two tailed *z*-test for independent pearson correlations was used.

### Results

As obvious in Figure 1 there is no difference for the HS-scores between women and men for neither of the pictures: picture A [*M*_men_ = 2.25, *SD*_men_ = 1.17, *M*_women_ = 2.15, *SD*_women_ = 1.35, *t*_(98)_ = 0.37, *p* = 0.71, *d* = 0.07], picture B [*M*_men_ = 0.53, *SD*_men_ = 0.91, *M*_women_ = 0.69, *SD*_women_ = 0.90, *t*_(98)_ = −0.92, *p* = 0.36, *d* = −0.19], picture C [*M*_men_ = 2.25, *SD*_men_ = 1.30, *M*_women_ = 2.42, *SD*_women_ = 1.19, *t*_(98)_ = −0.69, *p* = 0.49, *d* = −0.14], picture D [*M*_men_ = 1.10, *SD*_men_ = 0.98, *M*_women_ = 1.49, *SD*_women_ = 1.24, *t*_(98)_ = −1.68, *p* = 0.09, *d* = −0.34], picture E [*M*_men_ = 2.08, *SD*_men_ = 1.38, *M*_women_ = 2.22, *SD*_women_ = 1.23, *t*_(98)_ = −0.55, *p* = 0.59, *d* = −0.11], picture F [*M*_men_ = 1.45, *SD*_men_ = 1.20, *M*_women_ = 1.90, *SD*_women_ = 1.24, *t*_(98)_ = 0.45, *p* = 0.66, *d* = 0.09].

For FF there is also no difference of women and men for picture A [*M*_men_ = 0.35, *SD*_men_ = 0.48, *M*_women_ = 0.31, *SD*_women_ = 0.59, *t*_(98)_ = 0.08, *p* = 0.70, *d* = 0.02], picture B [*M*_men_ = 0.88, *SD*_men_ = 0.91, *M*_women_ = 1.05, *SD*_women_ = 1.15, *t*_(98)_ = 0.15, *p* = 0.42, *d* = 0.03], picture E [*M*_men_ = 0.48, *SD*_men_ = 0.08, *M*_women_ = 0.47, *SD*_women_ = 0.70, *t*_(98)_ = 0.09, *p* = 0.99, *d* = 0.02], picture F [*M*_men_ = 0.40, *SD*_men_ = 0.10, *M*_women_ = 0.34, *SD*_women_ = 0.60, *t*_(98)_ = 0.11, *p* = 0.08, *d* = 0.02]. But women yield higher scores of FF in picture C [*M*_men_ = 0.35, *SD*_men_ = 0.58, *M*_women_ = 0.75, *SD*_women_ = 1.06, *t*_(98)_ = 0.10, *p* = 0.03, *d* = −0.19] and picture D [*M*_men_ = 0.55, *SD*_men_ = 0.13, *M*_women_ = 1.03, *SD*_women_ = 1.05, *t*_(98)_ = 0.14, *p* = 0.02, *d* = 0.03].

#### Construct validity

The correlations between the two factors Hope of Success (HS) and Fear of Failure (FF) are with *r* = −0.50 (*p* < 0.01) in the male sample a little higher than in the female sample (*r* = −0.30, *p* < 0.05, *Z* = −1.14, *p* > 0.05). As reported in Table 3, the correlations between the implicit HS- and FF-scores and the two questionnaires SELLMO and TAT-Q are only in the male sample and only for FF in a moderate area from −0.55 to 0.51, all other correlations where not statistically significant and low. In the female sample, these correlations range from 0.01 to 0.21. Only the correlations between FF and achievement goals as well as the correlation between FF and avoidance goals (*r* = 0.29, *p* < 0.05) are statistically significant. For HS the independence between questionnaire-scores and TAT/PSE can also be attested in parts. Especially the correlations between the implicit HS and the SELLMO are high for women (*r* = 0.31–0.41) and men (*r* = −0.55–0.38). The relation between avoidance goals and HS (*r* = −0.55, *p* < 0.01) is for men lower than for women (*r* = 0.41, *p* < 0.05, *Z* = 3.76, *p* < 0.01). This result extends to the correlations between achievement goals and implicit HS, which is even lower in the male sample (*r* = −0.42, *p* < 0.01) than in the female sample (*r* = 0.32, *p* < 0.05, *Z* = 2.40, *p* < 0.05).

**Table 3:** **Discriminant validity of TAT/PSE with female pictures for men (*N* = 40) and women (*N* = 60)**.

	**Hope of success**				**Fear of failure**			
	**Men**	**Women**	***Z***	***p***	**Men**	**Women**	***Z***	***p***
SELLMO_LG	0.38^*^	−0.01	1.40	0.16	−0.26	0.21	0.66	0.51
SELLMO_AV	−0.42^**^	0.32^*^	2.40	0.02	0.41^**^	0.29^*^	1.30	0.19
SELLMO_VG	−0.55^**^	0.41^*^	3.76	0.00	0.51^**^	0.29^*^	1.19	0.23
SELLMO_AG	0.02	0.31^*^	1.05	0.29	0.38^*^	0.01	0.22	0.83
TAT-Q_HS	−0.45^**^	0.23	2.30	0.02	0.32^*^	0.11	0.51	0.61
TAT-Q_FF	−0.43^**^	0.07	1.79	0.07	0.17	0.01	0.77	0.44

SELLMO, Scales of learning and achievement motivation; LG, learning goals; AG, approach goals; VG, avoidance goals; AV, effort-avoidance goals; HS, hope of success; FF, fear of failure.

* p < 0.05,

** *p < 0.01*.

Additionally, relations between implicit motives and explicit motives, assessed using the self-reported questionnaire TAT-Q, can be reported. The HS-score correlates negatively with self-reported HS (*r* = −0.45, *p* < 0.01) in the male sample and positively in the female sample (*r* = 0.23, *p* > 0.05, *Z* = 2.30, *p* < 0.05). The correlation of HS and explicit FF was also moderate for men (*r* = −0.43, *p* < 0.01) but not for women (*r* = 0.07, *p* > 0.05, *Z* = 1.79, *p* = 0.07).

#### Criterion validity

As obvious in Table 4, differences between women and men can be reported. The expected correlations between HS and correct (medium-difficult) tasks are in the female sample not statistically significant (*r* = 0.03, *p* > 0.05) and in the male sample negatively (*r* = −0.50, *p* < 0.01, *r* = 0.03, *p* > 0.05; *Z* = 2.24, *p* < 0.05). The task distance is for both genders negatively related to HS (*r*_women_ = −0.23, *r*_men_ = −0.27, *Z* = 0.18, *p* > 0.05).

**Table 4:** **Criterion validity of TAT/PSE with female pictures for men (*N* = 40) and women (*N* = 60)**.

	**Hope of success**				**Fear of failure**			
	**Men**	**Women**	***Z***	***p***	**Men**	**Women**	***Z***	***p***
TCPT_CC	−0.50^**^	0.03	2.24	0.03	0.12	−0.02	0.03	0.98
TCPT_DC	−0.27	−0.23	0.18	0.86	−0.17	0.19	1.76	0.08
LT general	−0.43^**^	0.78^**^	5.49	0.00	0.64^**^	0.22	2.08	0.04
LT before tests	−0.40^*^	0.57^**^	3.07	0.00	0.58^**^	0.21	0.85	0.40
LT during holidays	−0.30	−0.11	0.55	0.58	−0.11	0.05	1.12	0.26
Overall grade	−0.20	0.35^*^	2.47	0.01	−0.23	−0.35^*^	0.50	0.62
QLB_positiv	−0.48^**^	0.19	1.88	0.06	0.34^*^	0.25	1.49	0.14
QLB_negativ	−0.27	−0.04	1.65	0.10	−0.01	−0.11	0.48	0.63

TCPT, Task-choice-performance-test; CC, correct choices; DC, distance of choices; LT, learning time; QLB, questionnaire of learning behavior.

* p < 0.05,

** *p < 0.01*.

According to the expectations learning time is positively associated with HS (*r* = 0.57, *p* < 0.01 for learning time before tests and *r* = 0.78, p < 0.01 for general learning time) in the female sample, but not in the male sample. Men with high HS spent less time in learning (*Z* = 5.49, *p* < 0.01) and learning before tests (*Z* = 3.07, *p* < 0.01). The same effect is visible for fear of failure (FF): women with high FF spent less time on learning (*r* = 0.22, *p* > 0.05) than men (*r* = 0.64, *p* < 0.01, *Z* = 2.08, *p* < 0.05).

Regarding the outcomes it is visible that men (*r* = −0.35, *p* < 0.05) as well as women (*r* = −0.23, *p* > 0.05) tend to get better grades, when they have high FF. But also men also show better grades, when they have high HS (*r* = −0.20, *p* > 0.05), while women do not (*r* = 0.35, *p* > 0.05, *Z* = 2.47, *p* < 0.05). The implicit HS and FF do not correlate with positive and negative learning behavior in the female sample. Men show more positive learning behavior when they are motivated by FF (*r* = 0.34, *p* < 0.05) rather than by HS (*r* = −0.48, *p* < 0.05). On the other hand women show more positive learning behavior when they have higher HS (*r* = 0.19, *p* < 0.05) and men (*r* = −0.48, *p* < 0.05, *Z* = 1.88, *p* = 0.06).

#### Brief discussion

The results show less difference between women and men, when the TAT/PSE consists of women in the pictures. Only the picture C and D women show more fear of failure, this effect could cause from the fact that here two women are portrait working together in the picture. For both samples neither the independence of HS and FF nor the independence of implicit and explicit motives could be verified. The test seems more face valid for women, because their TAT/PSE-scores correlate with questionnaire scores. Furthermore, the criterion validity is not satisfying. Neither task-choice performance nor learning behavior correlate with any of the motive-scores in the expected way in the male sample. But the modified women TAT/PSE showed relationship to learning time of women. A possible reason for the low validity could be, that women are less associated with achievement context.

## General discussion

The TAT/PSE of Heckhausen (1963) is often been criticized for it neglects women in the pictures. Therefore, it is said, women cannot project their own implicit motives onto the protagonists of the TAT pictures as men do, because in the pictures are only men presented. To assess if the gender of the protagonists really is important for the measurement of implicit achievement motive two studies were assessed. The picture of the first study included only male person in the picture, and the pictures of the second study only female protagonists. Katz et al. (1993) and Worchel et al. (1990) found that women and men do not differ in their motive-scores regardless if they see men or women in the pictures. In this research it could only particularly be attested: Men show more hope of success in pictures that should stimulate fear of failure by presenting a male authority (teacher, boss). Women, on the other hand show more fear of failure, when they see two women in the picture (girl and female teacher, two women in the laboratory), where one woman could probably be the authority of the other woman. For all other pictures there was no difference in HS and FF as it is also reported by Schultheiss and Brunstein (2001), Pang and Schultheiss (2005), Langan-Fox and Grant (2006), Tuerlinckx et al. (2002), and Stewart and Chester (1982). The reason for the gender-difference in the present study could also cause from the fact, that in all above cited studies the two components HS and FF were not separately assessed, the focus was only on the general achievement motive. So especially the different perception of an authority in the pictures is more contrasted in this study, probably the influence of gender-specific attitudes on implicit motive assessment could be important for further research.

A closer look at the validity showed little difference. The analysis of the Heckhausen TAT showed no difference in discriminant validity of women and men. So the independence of implicit and explicit motives could be attested, as Spangler (1992) as well as Köllner and Schultheiss (2014) found in their meta-analysis. Regarding the task-choice performance, the model of Atkinson (1957) could be particularly replicated in the fact that both men and women with high fear of failure avoided to choose medium difficult tasks. Furthermore, it is shown that a high HS-score is associated with more learning time, as it is assumed. The only gender-difference here was that women prefer learning during holidays and men only learn before tests. This could rather cause from gender-specific high studiousness in achievement-oriented women (Duckworth and Seligman, 2006) than from differences in validity. For both genders a positive effect of achievement motives on grades can be reported. For learning behavior it was shown that only men with high FF show negative learning behavior in the expected way (Chen et al., 2009; Haghbin et al., 2012; De Castella et al., 2013). So the validity of the original Heckhausen (1963) TAT/PSE can be attested in both genders.

The female version of the TAT did not measure according to the hypothesis. The independence of implicit and explicit motives could not be attested. Furthermore, the female version of the TAT seems to be more face valid for women, as their implicit motive-score correlates with questionnaires. Also the risk-choice behavior could not be predicted not for women and not for men. Contrary, it is shown that men with high HS avoid medium-difficult tasks. Also the correlation of learning behavior and implicit motives was against the hypothesis for men in that way that men with high HS spent less time in learning. This is surprising because Heckhausen (1963) constructed HS as an approaching component and as an implicit motive it should energize the behavior. Also it was shown that men have, against the hypothesis positive learning behavior if they have low HS and high FF. The correlations to grades on the other hand worked for women as well as for men. This shows that the modified version of Heckhausens TAT/PSE (1963) does not validly measure the achievement motive, especially for men. A possible reason therefore could be that for men women are not that strong associated with achievement situations than men and so implicit gender stereotypes confound the measurement of the achievement motive (Aronow et al., 2013). Especially the aspect of perceiving a woman or a man as authority could be important here. For further research it could be interesting to present both genders in a picture and analyse how this influences the achievement motive score. Another problem is that for study 1 and 2 different samples are used. Although they were comparable regarding demographical variables, this could also have influenced the results.

## Ethics statement

It was not necessary for the study for there was no ethics relevant problems. People just filled out questionaires/tests, afterwards they got feedback on their scores. No manipulation or violation was done.

## Author contributions

NG designed the experiments, performed the experiments, analyzed the data and wrote the article.

### Conflict of interest statement

The author declares that the research was conducted in the absence of any commercial or financial relationships that could be construed as a potential conflict of interest. The reviewer MP and handling Editor declared their shared affiliation, and the handling Editor states that the process nevertheless met the standards of a fair and objective review.
